# Enteral Nutrition in Idiopathic Parkinson's Disease and Atypical Parkinsonism: A Systematic Review

**DOI:** 10.1002/mdc3.70653

**Published:** 2026-05-01

**Authors:** Bradley Lonergan, Matteo Ciocca, Anette Schrag, Yen Tai

**Affiliations:** ^1^ Department of Brain Sciences, Charing Cross Hospital, Hammersmith Imperial College London London UK; ^2^ Department of Neurology, Charing Cross Hospital, Hammersmith Imperial College Healthcare Trust (ICHT) London UK; ^3^ Department of Clinical and Movement Neurosciences, UCL Queen Square Institute of Neurology University College London London UK

**Keywords:** enteral feeding, gastrostomy, parkinsonism, survival

## Abstract

**Background:**

Dysphagia and malnutrition are common in advanced Parkinson's disease and atypical parkinsonism. There is a lack of evidence to guide the use of enteral nutrition in these situations, including whether it improves survival or reduces aspiration pneumonia.

**Objectives:**

To systematically review the impact of enteral nutrition in Parkinson's disease and atypical parkinsonism on survival and morbidity (including aspiration pneumonia).

**Methods:**

We followed PRISMA guidelines and conducted searches using PubMed and Scopus databases. ROBINS‐I V2 (*n* = 2) and JBI Cohort Study (*n* = 14) checklists were used to assess risk of bias.

**Results:**

We identified 16 eligible studies. Risk of bias was moderate or high in all studies. Reported enteral feeding rates in parkinsonian disorders varied widely. The rate of aspiration pneumonia following enteral feeding was around 40% in those with parkinsonism. The weighted median survival following gastrostomy insertion in Parkinson's disease was 1.35 years (*n* = 83; 3 studies) and in atypical parkinsonism 1.49 years (*n* = 46; 2 studies). One study in atypical parkinsonism found improved survival with enteral feeding compared to at‐risk feeding (24 vs. 12 months), whilst another in Parkinson's disease found no difference in mortality rates.

**Conclusions:**

There is a lack of high‐quality evidence on the impact of enteral feeding on prognosis and morbidity. It is unclear whether enteral feeding improves survival compared to at‐risk feeding. There is a need for more robust evidence such as through enteral feeding registries with standardized data collection.

Dysphagia affects over 80% of people with idiopathic Parkinson's disease (PD) and atypical parkinsonism (e.g. Progressive Supranuclear Palsy [PSP]; Multiple System Atrophy [MSA]) during their lifetime.^1^, ^2^ The median time from diagnosis to development of dysphagia is shorter for PSP (42 months) and MSA (67 months) than PD (~130 months).^1^ Dysphagia is a poor prognostic marker across parkinsonian subtypes, as it leads to aspiration pneumonia and malnutrition, which are the commonest reasons for hospitalization and mortality in PD and PSP.^2^, ^3^, ^4^ It is also associated with increased psychosocial burden, anxiety and depression.^5^, ^6^, ^7^, ^8^ Despite this, dysphagia is not included in most quality‐of‐life questionnaires for parkinsonism (e.g. PDQ‐39, EQ‐5D) and a validated screening tool has only recently been recommended for use in PD.^9^

The mainstay of treatment for dysphagia is conservative measures, such as: only eating in medication‐ON (for PD); eating when fully alert; eating whilst positioned upright; and altering food consistency.^10^ Studies exploring the impact of levodopa on dysphagia in PD have shown mixed results; some show no effect and others show only modest improvements.^11^, ^12^, ^13^ There have been case reports of improvement of dysphagia in PSP with levodopa, but it is generally considered non‐levodopa responsive.^14^ Alternatively, enteral feeding can be considered, as a more invasive option to prevent malnutrition.^2^, ^15^, ^16^ Throughout this article, the term ‘enteral feeding’ refers to long‐term feeding via any route (ie, Percutaneous Endoscopic Gastrostomy [PEG], Radiologically Inserted Gastrostomy [RIG], Nasogastric [NG]).

The evidence base for enteral feeding is low quality across indications, given the retrospective and uncontrolled studies available, but professional recommendations vary based on this evidence. For example, enteral feeding is recommended for Amyotrophic Lateral Sclerosis (ALS)^17^, ^18^ and stroke.^19^ By contrast, it is strongly discouraged for advanced dementia and a Cochrane review found no evidence of benefit.^20^, ^21^, ^22^, ^23^, ^24^, ^25^ Enteral feeding recommendations in parkinsonism are less well‐defined, potentially due to clinical differences between PD and atypical parkinsonism and the heterogeneity of PD progression.^26^ Atypical parkinsonism has more rapid disease progression and earlier milestone accumulation than PD, as demonstrated in Table 1, meaning that enteral feeding decisions are required earlier and with greater urgency.^27^, ^28^ ALS is included in Table 1 to contrast with MSA and PSP; they are all rapidly progressive neurological conditions, but enteral feeding is more strongly recommended in ALS. This low‐quality evidence base and subsequent uncertainty contributes heavily to variation in practice worldwide, meaning that clinician and patient perspectives play a bigger role than for interventions with a stronger evidence base (e.g. deep brain stimulation).

**TABLE 1:** Summary of clinical features and milestones for several long‐term neurological conditions

	PD	PSP	MSA	ALS
Mean age at disease onset (years)	61–72^29^, ^30^	65^28^	56^31^	58–63^32^
Median disease duration to dysphagia (months) * Mean duration (months)	130^3^	42	67^3^	23*^33^ *Note: often present at diagnosis in bulbar ALS*
Median prognosis from diagnosis (years)	5–17^26^, ^34^	7–8.7^28^, ^35^	7.5–9.5^35^, ^36^	4^37^
Dementia prevalence (%)	47^38^	10–52^39^, ^40^	11^41^	15^42^

Abbreviations: ALS, Amyotrophic Lateral Sclerosis; MSA, Multiple System Atrophy; PD, Parkinson's disease; PSP, Progressive Supranuclear Palsy.

Personal values, such as religiosity and conservatism, are associated with a person's preferences for enteral feeding.^43^, ^44^ This probably explains why patients’ early opinions regarding enteral feeding predict subsequent uptake.^45^ Personal factors play an equally important role in clinicians’ decision‐making, as cultural norms and prior experiences probably affect their clinical approach. Surveys show that around half of People with Parkinson's disease (PwP) and their caregivers, and those with PSP, would not accept enteral feeding if offered it, with some geographical variation.^46^, ^47^, ^48^ However, the reasons why those with parkinsonism choose to accept or reject enteral feeding have not been explored. In ALS, patient‐centric (e.g. previous experience and beliefs), caregiver‐related (e.g. whether caregivers are involved in decision‐making or not) and clinician‐related (e.g. providing information, influencing decisions) factors are important in patients’ decision‐making on enteral feeding.^44^, ^49^, ^50^ It is feasible that some of these factors also apply to parkinsonism, but further qualitative research is needed to confirm this.

Although the ethical and legal frameworks of enteral feeding decision‐making have been well explored, real‐world implementation is often complex.^51^, ^52^, ^53^ For example, a third of people referred for PEG insertion lack capacity and are unable to express their preferences.^51^ Despite Advanced Care Planning (ACP) being included in national guidelines for PD, only 23% of parkinsonian patients undergoing PEG had any ACP in place and it is unlikely that all of these discussions include feeding specifically.^54^, ^55^, ^56^ In situations where a PwP lacks capacity (e.g. moderate–severe dementia) and has no ACP, which anecdotally seems to be common, clinicians lead decision‐making in their best interests. This individualized and holistic decision‐making process should consider the risks and benefits of PEG insertion and enteral feeding, informed by the perspectives of the MDT, the caregiver and any previously expressed wishes.

The outcomes from long‐term enteral nutrition have been poorly explored in PD and atypical parkinsonism, making it difficult for clinicians to accurately communicate the potential risks and benefits to patients. A lack of evidence is the most cited reason by clinicians for not recommending enteral nutrition in atypical parkinsonism.^57^ The aims of this systematic review are to: (a) summarize the mortality and long‐term complications data for enteral feeding (including related to PEG insertion) in parkinsonism; (b) summarize the rate of parkinsonism as an indication for PEG insertion in an unselected endoscopy population; (c) evaluate the available guidance from relevant international societies and published reviews on initiating enteral feeding in parkinsonism.

## Methods

The authors conducted a systematic review following the Preferred Reporting Items for Systematic Reviews and Meta‐Analyses (PRISMA) guidelines. PubMed and Scopus databases were used without filters. The following search terms were used (including the Boolean terms “AND” and “OR”): “parkinson*,” “Multiple System Atrophy,” “Progressive Supranuclear Palsy,” “Corticobasal Degeneration,” “tube feeding,” “artificial feeding,” “artificial nutrition,” “nutrition,” “feeding perspective*,” “feeding,” “gastrostomy,” “gastrostomy feeding”, “jejunostomy.”

The inclusion criteria were studies in patients with PD and/or atypical parkinsonism with outcome data on enteral feeding. Outcomes included were: (a) mortality rates; (b) aspiration pneumonia; (c) non‐specific “complications” (short‐ and long‐term); (c) weight changes; (d) latency from diagnosis to enteral feeding.

The Risk Of Bias In Non‐randomized Studies—of Interventions, Version 2 (ROBINS‐I V2) assessment tool was used where there was a comparator group and the JBI Cohort Study checklist was used for studies without a comparator group.

We also identified papers presenting (a) the frequency of PD or atypical parkinsonism as PEG indication from general adult population endoscopy services (Table S1); (b) the frequency of enteral feeding in PD and/or atypical parkinsonism cohorts (Table S2); (c) recommendations (e.g. expert opinion, consensus statement, review) for/against PEG or PEG timing in PD and/or atypical parkinsonism patients (Table S3). More detailed information about these papers are found in Table S4 and S5.

To provide a sense of evidence strength, result averages are described using the following: n = the total number of patients in the category; the total number of studies which provided these patients. Weighted averages were calculated by multiplying the mean or median (e.g. latency, mortality) by the number of patients in that study, so that studies including more patients are more heavily weighted for the overall averages.

## Results

**Figure 1:**
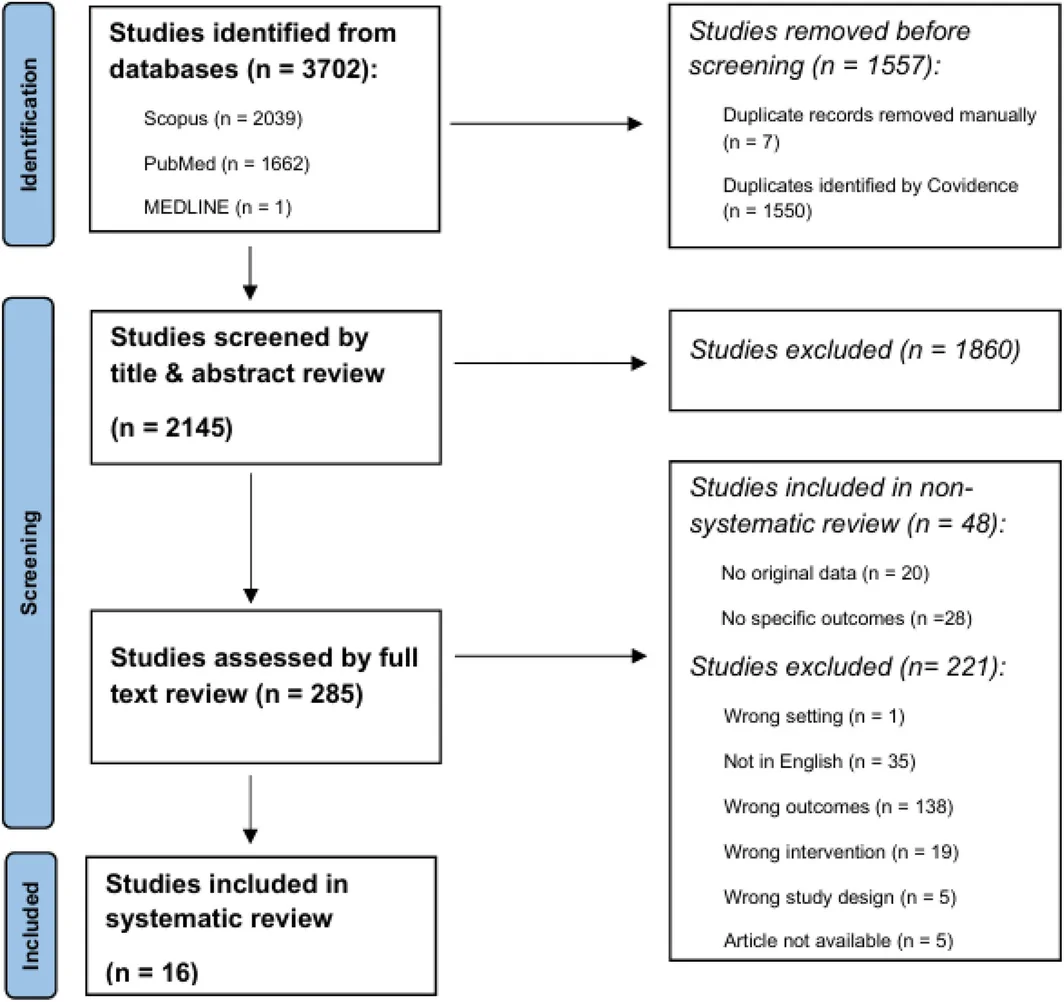
PRISMA flowchart for selection of included studies in this systematic review.

From a total of 3702 papers, 1557 duplicates were removed. A further 1860 papers were excluded after title and abstract review, and 269 papers were excluded after full text review, most commonly due to containing the wrong outcomes. Thus, 16 papers were included in the systematic review of outcomes (see Fig. 1 and Table 2 and 3). Two of these studies had a comparator group (ie, at‐risk oral vs enteral feeding) (Supplementary Table 6 4, 58;) and 14 did not (Supplementary Table 7 54, 59, 60, 61, 62, 63, 64, 65, 66, 67, 68, 69, 70, 71;). No randomized controlled trials were found. The overall quality of these studies included was therefore grade III and below (Fig. 1).

**TABLE 2:** Summary of mortality, long‐term complication (including aspiration pneumonia) and latency data in Parkinson's disease

Outcome	Lang et al 2004	Malmgren et al 2011	Yamazaki et al 2011	Goh et al 2016	Kara et al 2016	Sarkar et al 2017^a^, ^b^	Marois et al 2017^a^	Brown et al 2020^a^	Tiankanon et al 2022^d^	Weighted averages^c^
Patient groups	PD	PD > 65 y/o	PD	PD (with VFS)	PD > 60 y/o	PD/MSA/PSP	PD/MSA/PSP/CBD/DLB	PD/PSP/MSA/LBD/vascular	PD	
Country	Israel	Sweden	Japan	Singapore	Turkey	UK	France	UK	Thailand	
Number of participants with PEG (% PD)	28	11	8	34	29	13 (n/a)	32 (22% PD)	93 (70% PD)	42	
30 day mortality post‐PEG (%)	6	9				8		6.9 (PD)		6.9
1 year mortality (%)		55					59.4			56.7
2 year mortality (%)							65.6			
5‐year mortality (%)					72.4					
Median survival after PEG (years)		0.64				1.20	0.50	1.56 (PD)		1.35
Mean survival after PEG (years)						1.25 (PD)				
Latency from onset to PEG insertion (years)						16.2 (PD)				
Aspiration pneumonia at any time following PEG (%)				61.8		23	46	34		43.7
Long‐term (>30 days) complication (%)						69	69		33	
Weight change 1‐year after PEG (Kg)			−3							

Abbreviations: ALS, Amyotrophic Lateral Sclerosis; MSA, Multiple System Atrophy; PD, Parkinson's disease; PSP, Progressive Supranuclear Palsy; VFS, videofluoroscopy.

^a^ For studies which included a combination of diagnoses, included statistics refer to the overall cohort of patients (unless otherwise stated).

^b^ Excluded from weighted averages, as patient groups (e.g. PD, PSP) are undifferentiated or statistics for whole group not available.

^c^ Weighted averages are combined for all atypical parkinsonism cases, unless otherwise stated.

^d^ In this study, “long‐term” is classified as >6‐months.

**TABLE 3:** Summary of mortality, long‐term complication (including aspiration pneumonia) and latency data in atypical parkinsonism

Outcome	Goetz et al 2003	Nath et al 2003	Sarkar et al 2017^a^, ^b^	Marois et al 2017^a^	Brown et al 2020^a^	Do et al 2020	Homma et al 2020	Mahale et al 2021	Kobylecki et al 2024	Wrigley et al 2025	Weighted averages^c^
Patient groups	PSP	PSP	PD/MSA/PSP	PD/MSA/PSP/CBD/DLB	PD/PSP/MSA/LBD/vascular	MSA	MSA	PSP	MSA/PSP/CBS	PSP/LBD/CBD/MSA	
Country	USA	UK	UK	France	UK	Korea	Japan	India	UK	UK	
Number of participants with PEG *Per disease group*	27	16	13 (n/a)	32 (22% PD) *7/15/5/3/2/8*	83 (70% PD) *58/10/5/3/7*	25	9	34	21 (52% PSP) *7/11/3*	177 (86% PSP) *152/6/5/8*	
30‐day mortality post‐PEG (%)			8		10 (PSP); 0 (other non‐PD)						
1‐year mortality (%)				59.4							
2‐year mortality (%)				65.6							
Median survival after PEG (years)		4.1^b^ (latency <3 yrs) 9.6 (latency >3 yrs)	1.20	0.50^b^	1.06 (all) 1.10 (PSP); 1.16 (MSA); 1.06 (vasc); 0.78 (LBD)				2 (1 no PEG)		1.49
Mean survival after PEG (years)			1.25				5.94 (MSA‐B 11.5, MSA‐A 1.50)				
Latency from onset to PEG insertion (years)	7.25	5	6.3 (MSA); 6.5 (PSP)	8.5 (MSA)		7		5.6	6 (MSA); 6.7 (PSP); 5.9 (CBS)	6.8 (PSP); 8.2 (LBD); 3.9 (CBD); 4.5 (MSA)	6.9 (MSA) 6.6 (PSP)
Aspiration pneumonia at any time following PEG (%)			23	46	34						39.7
Long‐term (>30 days) complication (%)			69	69							

^a^ For studies which included a combination of diagnoses, included statistics refer to the overall cohort of patients (unless otherwise stated).

^b^ Excluded from weighted averages, as patient groups (e.g. PD, PSP) are undifferentiated or statistics for whole group not available.

^c^ Weighted averages are combined for all atypical parkinsonism cases, unless otherwise stated.

Of the 269 papers excluded from the systematic review, 48 are non‐systematically discussed, as they provide wider context on this subject regarding: (a) frequency of PD or atypical parkinsonism as PEG indication (Table S1 65, 66, 67, 72, 73, 74, 75, 76, 77, 78, 79, 80, 81, 82, 83, 84, 85, 86, 87, 88, 89); (b) frequency of enteral feeding in PD and/or atypical parkinsonism cohorts (Table S2 59, 62, 64, 90, 91, 92, 93, 94, 95); (c) recommendations on PEG placement in parkinsonism (Table S3 and S5 2, 10, 16, 56, 95, 96, 97, 98, 99, 100, 101, 102, 103, 104, 105, 106, 107, 108, 109, 110, 111, 112).

### Frequency of Enteral Feeding in Parkinsonism

Parkinsonism (predominantly PD) was listed as the indication for gastrostomy or jejunostomy insertion in 2.7–37.0% (weighted average 5.1%) patients across endoscopy departments in 21 studies (Table S1). The prevalence of PEG feeding in patients with parkinsonism was highly variable, ranging from <5% (PD without dementia in USA 1985–1990) to 64% (MSA in Japan 2000–2014) across nine studies (Table S2 59, 62, 64, 90, 91, 92, 93, 94, 95). Enteral feeding rates were reported to be growing in some countries (e.g. Taiwan) and falling in others (e.g. USA).^78^, ^90^, ^95^

### Mortality Data

Table 2 and 3 describe the 17 studies included in this systematic review with participants with PD (six studies; Table 2), atypical parkinsonism (seven studies; Table 3) or a combination of the two (three studies; included in both Table 2 and 3).

The weighted average mortality after PEG insertion in PD (excluding the studies that did not differentiate PD and atypical parkinsonism) was 6.9% at 30 days (*n* = 104; three studies, range 6–9%) and 56.7% at 1 year (*n* = 18; two studies, range 55–59.4%) (Table 2). Median survival was 1.35 years post‐PEG insertion (*n* = 83; three studies, range 0.50–1.56 years). For atypical parkinsonism (Table 3), 30 day and 1 year mortality rates were comparable to PD but often included those with PD. The weighted median post‐PEG survival was 1.49 years (*n* = 46; two studies, range 0.78–2 years) in atypical parkinsonism. In one study, prognosis appeared to be worst in Lewy body dementia (0.78 years; *n* = 3), though 30 day mortality was highest for PSP (10%, *n* = 10).^54^ More detailed mortality data, from these studies and others, (including 3 month, 6 month, 2 year and 5 year post‐PEG) and are provided in Table S4.

One study found that survival was greater in those with atypical parkinsonism who received enteral feeding (24 months) than those in whom gastrostomy was not performed (12 months) and received at‐risk oral feeding.^58^ This remained significant when those already receiving enteral feeding before the start of the retrospective study were removed. In PD, long‐term mortality rates (from aspiration pneumonia) was slightly higher in the enteral feeding group (33%) than the risk feeding group (21.4%), though this was not significant.^4^

Regarding predictors of worse survival, one study in patients with PD, MSA, PSP, corticobasal degeneration or Lewy body dementia showed significantly worse survival rates in those who were completely dependent on others to complete their activities of daily living.^63^ Although advanced dementia is a recognized contraindication for enteral feeding, survival rates after PEG insertion in PwP with (344 days) and without (584 days) dementia were not significantly different in one study.^54^ Although this study did not formally rate dementia severity, the authors concluded that some patients (e.g. milder dementia) may be suitable for enteral feeding if their symptoms are levodopa‐responsive.

### Morbidity and Complication Data

Aspiration pneumonia at any time following PEG was 43.7% in PD (*n* = 106; range 34–61.8%; Table 2 4, 54, 63;) and 39.7% in atypical parkinsonism (*n* = 50; range 34–46%; Table 3 54, 63), demonstrating that the risk of aspiration pneumonia is not abolished post‐PEG insertion. Mean long‐term (> 30 days) complication rate was 69% in undifferentiated parkinsonism patients post‐PEG (*n* = 45; two studies) and was significantly lower in one PD‐only study (40%^69^). One retrospective study reported a trend towards lower admission rates with aspiration pneumonia in PD patients with dysphagia who opted for enteral feeding (61.8%) over risk feeding (66.7%).^4^

Two studies suggested that parkinsonian patients with PEG are less likely to live independently than those without. Brown et al 2020 found that 32% of patients with parkinsonism admitted from home were institutionalized on discharge after PEG insertion.^54^ Furthermore, various studies show a higher rate of interaction with healthcare, namely by hospital readmission, following PEG insertion. 47% of PD patients were readmitted in the year after PEG insertion in the study by Brown et al 2020.^54^ Kim et al 2021a show a greater than double chance of living in a long‐term facility in those with PEG than those without in PD.^95^ The median length of hospital stay was double in those with a PEG (9.5 days) than those without (4 days).^95^ This study was not included in the systematic review, as the exact study population size (ie, those with PEG) was unclear.

Despite weight loss being a common indication for initiation of enteral feeding, it is not clear that enteral feeding has any long‐term impact on weight loss in PD.^57^ Only one study reported changes to weight following enteral feeding, finding no significant change to weight (3 kg loss) after significant weight loss in the 12 months prior to enteral feeding initiation (8 kg loss).^71^ A Cochrane review highlighted the “*lack of robust evidence”* for enteral feeding on weight loss, though PD patients were only included in a mixed patient cohort.^96^

### Timing of PEG Insertion

The median latency from disease onset to PEG insertion were as follows: PD 16.2 years (*n* = n/a^61^), MSA 6.9 years (*n* = 55^58^, ^62^, ^63^, ^68^) and PSP 6.6 years (n = 240^58^, ^59^, ^60^, ^68^, ^70^) (Table 2 and 3). These figures are weighted for PSP and MSA.

The effects of PEG insertion timing/latency were discussed. Nath et al 2003 found a trend towards higher mortality in those who had early PEG insertion in PSP.^60^ However, this was an observational study and early swallowing problems, a significant confounder representing more aggressive disease progression, were also associated with significantly shorter survival.^3^

### Expert Opinions and Consensus Statements on Enteral Feeding for Parkinsonism

Consensus statements and expert recommendations regarding enteral feeding in parkinsonism were made in 22 studies (Table S3). The most common recommendations were for an individualized approach, given the lack of robust evidence in favor of enteral feeding. The complete breakdown of recommendations is as follows: studies advocating an individualized approach due to a lack of evidence,^2^, ^10^, ^16^, ^56^, ^96^, ^97^, ^98^, ^99^, ^100^ without PEG specific guidance for parkinsonism,^110^, ^111^, ^112^ highlighting the importance of ACP,^104^, ^105^, ^106^ advising avoiding enteral feeding due to high dementia prevalence in advanced PD,^95^, ^109^ advocating enteral feeding as a “last resort”^101^, ^102^ or only suitable for a minority of patients,^103^ highlighting lack of efficacy (e.g. on aspiration pneumonia, quality of life) of enteral feeding,^108^ avoiding enteral feeding in older patients/those with many co‐morbidities^56^ and recommending enteral feeding should be considered (in advanced PSP^107^).

There is some disagreement within the literature about the impact of enteral feeding on risk of aspiration. Some propose that enteral feeding reduces rates of aspiration pneumonia,^2^, ^105^ whilst others suggest that it *“does not prevent”* aspiration pneumonia (in PD^106^, ^108^; in MSA^99^). Another acknowledges that this is a controversial area without consensus in any patient group.^110^

Expert reviews and consensus statements generally mention a lack of evidence (in PSP^97^) and high uncertainty (in MSA^99^) regarding timing of PEG feeding in parkinsonism. Other groups recommended PEG insertion when patients can no longer meet nutritional needs (PSP^107^; PD: Schindler et al 2021; General^110^) or proposed PEG insertion soon after development of aspiration pneumonia (in PSP^2^).

## Discussion

This systematic review found a lack of high‐quality evidence to support, or refute, the use of enteral feeding in parkinsonism, including impact on mortality or morbidity (e.g. aspiration pneumonia). This at least partly explains the variability in enteral feeding rates between studies in patients with parkinsonism and supports the most common expert consensus recommendation to take an individualized approach using clinical judgment. Median survival following enteral feeding initiation is poor in PD (1.35 years) and atypical parkinsonism (1.49 years), but there are a lack of studies using comparator groups so the impact of confounding factors (e.g. disease progression) cannot be easily accounted for and the risk of bias is high (Table S6 and S7). The lack of evidence makes this a key research area for those with parkinsonism.^9^ Additionally, those who are most frail and have the worse prognosis would not be offered enteral feeding and would instead adopt oral at‐risk feeding. Thus, the data presented here relates to those patients well enough to proceed with enteral feeding and not the wider cohort of parkinsonian patients with dysphagia.

### Effect of Enteral Feeding on Mortality

Survival after initiating enteral feeding in PD (1.35 years) and atypical parkinsonism (1.49 years) seems to be comparable with ALS (weighted average 1.43 years; 110 patents)^113^, ^114^ and greater than in advanced dementia (weighted average 0.52 years; 145 patients, three studies).^115^, ^116^, ^117^ Dysphagia is an important milestone of progression in these conditions, which is an important confounder for studies of enteral feeding which is difficult to account for, particularly without randomization. Although one study found no difference between PD patients with and without dementia on survival, this does not necessarily contradict existing recommendations but support individualization of decision‐making. PwP with milder dementia in parkinsonism may be suitable candidates for enteral feeding, so severity of dementia should be accounted for in future studies.^24^, ^54^

Increased physical dependence at baseline was associated with worse survival from enteral feeding.^63^ This is probably a generalized prognostic factor which represents frailty, rather than a factor specific to parkinsonism, and an important confounder for studies of mortality after enteral feeding.

### Effect of Enteral Feeding on Morbidity

Rates of aspiration pneumonia remain high after initiating enteral feeding in PD (43.7%) and atypical parkinsonism (39.7%). Only one study compared enteral feeding with risk‐feeding directly, finding non‐significant reductions in aspiration‐related admissions.^4^ Overall long‐term complication rates are high, ranging from 33 to 69%. Despite quality‐of‐life playing an important role in enteral nutrition decision making, none of the studies in this review included quality‐of‐life as an outcome measure.

PwP were less likely to live independently following enteral feeding initiation and more likely to have longer hospital stays and readmission to hospital.^54^, ^95^ These risks should be included in shared decision‐making discussions, as institutionalization is may not an acceptable risk to some PwP, given that many PwP prefer to die at home.^118^ Older adults were more than twice as likely to develop chronic complications following PEG insertion, in a mixed patient cohort which included PD.^81^ More data is needed to understand the impact of enteral feeding on weight loss in PD and to make stronger conclusions.^71^, ^96^

### Timing of Enteral Feeding

PEG insertion occurs much earlier following diagnosis with MSA (6.9 years) or PSP (6.5 years) than with PD (16.2 years), though there is significantly less available evidence for PD. However, given the rapid disease progression with MSA and PSP, this is still relatively late in the disease course. This is demonstrated by the shorter latencies and prognoses in these conditions (see Table 1). The optimal timing for enteral nutrition in both PD and atypical parkinsonism is unclear. Although one study found worse survival with an early approach to PEG insertion (defined as <3 years after diagnosis) in PSP, this is probably confounded by worse disease severity (e.g. severe dysphagia) and/or rate of progression.^60^ Additionally, PSP subtype was not described; certain subtypes (e.g. Richardson syndrome) are more aggressive and associated with worse prognosis, regardless of the interventions implemented.^28^

In a survey of clinicians working with atypical parkinsonism, the preferred timing for enteral feeding was recurrent chest infections, > 10% weight loss in 6 months and persistent dysphagia, respectively.^57^ Although these tend to be later features, this study demonstrates that real‐world decision making utilizes clinical features more than disease duration alone. By contrast, a study in ALS patients demonstrated that early PEG insertion (weight loss <5 kg before PEG) had a 6 month better survival time than later PEG (weight loss >5 Kg).^119^ Atypical parkinsonism and ALS share some clinical features (see Table 1), but it is unclear whether clinical approaches to enteral feeding timing in these disease groups should be the same.

### Comparison to Other Neurodegenerative Disorders

There have been no previous systematic reviews on enteral feeding in parkinsonism. By contrast, Cochrane reviews have been conducted to summarize the evidence for enteral feeding in ALS^120^ and advanced dementia.^24^ Despite ALS, dementia and parkinsonism all being common indications for enteral feeding in real‐world practice, there are no randomized controlled trials comparing it with oral feeding at‐risk for any of these disease groups. Regarding controlled non‐randomized studies, there have been 14 in severe dementia, 23 in ALS and 2 in parkinsonism.^4^, ^24^, ^58^, ^120^

The studies in parkinsonian disorders had diverging results; one reported significantly improved survival with enteral feeding^58^ and one reported no differences in survival or aspiration pneumonia rates.^4^ There are also discrepancies in the recommendations made by professional bodies for these conditions. Although the quality of evidence is low, professional bodies make strong recommendations for enteral feeding in ALS^18^ and against enteral feeding in advanced dementia.^21^, ^24^ In parkinsonian disorders, where there are fewer research studies, an individualized approach has been recommended most.^111^

### Recommendations and Future Research

Given that randomized controlled trials of enteral feeding in parkinsonism are unlikely to occur, due to ethical constraints and the emotive nature of feeding decisions, we recommend the creation of national and/or international enteral feeding registries. This would allow collection of standardized baseline (e.g. indication, disease duration, objective dysphagia severity) and outcome data (e.g. 30 day and 1 year mortality, aspiration pneumonia incidence) to build a stronger evidence base. Those patients who opt for at‐risk oral feeding could also be enrolled, to provide a real‐world comparison of patients at a presumably similar disease stage. Although age was associated with worse outcomes in a mixed patient cohort,^81^ dependence and frailty are more specific factors and we hypothesize that they are stronger predictors of enteral feeding outcome. We recommend the routine measurement of key clinical factors (e.g. dementia, frailty) and their severity in clinical decision‐making and ongoing research.

We should consider the broader context in which feeding decisions are made, beyond mortality and morbidity rates. Defining what constitutes a “successful” outcome and, what quality of life means to an individual PwP, is important in real‐world practice. Few studies have explored the relationship between enteral nutrition and quality of life; no study in this review included quality of life measures as outcomes. Given that a third of people referred for PEG insertion lack capacity, mostly due to acute illness, ACP is vitally important to explore PwP's preferences (including feeding) before acute illness or irreversible cognitive changes occur.^51^ Future work should explore the perspectives of PwP and their carers regarding enteral feeding, as has been done more extensively in ALS.^49^, ^50^ New decision‐making tools are also needed, to support PwP in making and documenting these complex feeding decisions, similar that available via the Les Turner ALS Foundation.

## Conclusion

There is a lack of high‐quality evidence regarding the impact of enteral feeding on prognosis and morbidity in those with parkinsonism. Additionally, there is no consensus on which patient‐related factors should trigger initiation of enteral feeding or at which point in the disease process it should happen. The risk of institutionalization, high complication rates (including hospitalization and prolonged length of stay) and a lack of improvement in weight or aspiration pneumonia rates with enteral feeding should be mentioned as possible risks during decision‐making discussions, as some PwP may find these unacceptable risks. Creating a national and/or international enteral feeding registry for parkinsonism, with standardized data collection, is a potential pragmatic solution to the lack of evidence.

## AUTHOR CONTRIBUTIONS

**Bradley Lonergan:** Conceptualization; methodology; writing – original draft; writing – review and editing; data curation; formal analysis; project administration. **Yen Tai:** Conceptualization; writing – review and editing; supervision; methodology. **Anette Schrag:** Supervision; writing – review and editing. **Matteo Ciocca:** Writing – review and editing; supervision.

## Author Roles

Manuscript Preparation: **A**. Conception; B. Database search & literature review; C. Writing of the first draft; D. Review and Critique.

BL: 1A, 1B, 1C, 1D.

MC: 1D.

AS: 1D.

YT: 1A, 1D.

## Disclosures

Ethical Compliance Statement: The authors confirm that the approval of an institutional review board was not required for this work. We confirm that informed consent was not required, as this work did not involve any human subjects. We confirm that we have read the Journal's position on issues involved in ethical publication and affirm that this work is consistent with those guidelines.

Financial Disclosures and Conflicts of Interest: No specific funding was received for this work. The authors declare that there are no conflicts of interest relevant to this work.

Financial Disclosures for the previous 12 months: AS has received research funding or support from University College London, National Institute of Health Research (NIHR), NIHR ULCH Biomedical Research Centre, the International Parkinson and Movement Disorder Society (IPMDS), the European Commission and Parkinson's UK. She has received honoraria for consultancy from Biogen, Abbvie, Bial, Otsuka and Lilly. She has received royalties from Oxford University Press.

## Supporting information


**Table S1.** Frequency of parkinsonism as indication for PEG insertion in unselected/general endoscopy lists (*n* = 21)
**Table S2.** Prevalence of enteral feeding in parkinsonism groups (*n* = 9)
**Table S3.** Summary of recommendations regarding enteral nutrition in PD and atypical parkinsonism (*n* = 22)
**Table S4.** Detailed mortality and morbidity data after enteral feeding initiation (*n* = 14)
**Table S5.** Detailed recommendations on PEG placement in parkinsonism; ordered according to overall view towards PEG placement (*n* = 22)
**Table S6.** Risk of bias assessment for studies with a comparator group, using The Risk Of Bias In Non‐randomized Studies – of Interventions, Version 2 (ROBINS‐I V2) assessment tool (*n* = 2)
**Table S7.** Risk of bias assessment for studies without a comparator group, using the JBI Cohort Study checklist (*n* = 14)

## Data Availability

Data sharing not applicable to this article as no datasets were generated or analysed during the current study.

## References

[1] Coates C , Bakheit A . Dysphagia in Parkinson's disease. Eur Neurol 1997;38(1):49–52. 10.1159/000112902. 9252799

[2] Litvan I , Mangone CA , McKee A , et al. Natural history of progressive supranuclear palsy (Steele‐Richardson‐Olszewski syndrome) and clinical predictors of survival: a clinicopathological study. J Neurol Neurosurg Psychiatry 1996;60(6):615–620. 10.1136/jnnp.60.6.615 PMID: 8648326. PMC1073943 8648326

[3] Müller J , Wenning GK , Verny M , Mckee A , Chaudhuri KR , Jellinger K , et al. Progression of dysarthria and dysphagia in postmortem‐confirmed parkinsonian disorders. Arch Neurol 2001;58:259–264. 10.1001/archneur.58.2.259. 11176964

[4] Umemoto G , Furuya H . Management of dysphagia in patients with Parkinson's disease and related disorders. Intern Med Japanese Society of Internal Medicine 2020;59(1):7–14. 10.2169/internalmedicine.2373-18. PMC6995701 30996170

[5] Nath U , Thomson R , Wood R , Ben‐Shlomo Y , Lees A , Rooney C , Burn D . Population based mortality and quality of death certification in progressive supranuclear palsy (Steele‐Richardson‐Olszewski syndrome). J Neurol Neurosurg Psychiatry 2005;76(4):498–502. 10.1136/jnnp.2004.039370. PMC1739606 15774434

[6] Goh KH , Acharyya S , Ng SYE , Boo JPL , Kooi AHJ , Ng HL , et al. Risk and prognostic factors for pneumonia and choking amongst Parkinson's disease patients with dysphagia. Parkinsonism Relat Disord 2016;29:30–34. 10.1016/j.parkreldis.2016.05.034. 27321989

[7] Manor Y , Balas M , Giladi N , Mootanah R , Cohen JT . Anxiety, depression and swallowing disorders in patients with Parkinson's disease. Parkinsonism Relat Disord 2009;15(6):453–456. 10.1016/j.parkreldis.2008.11.005. 19071054

[8] Schrag A , Selai C , Davis J , Lees AJ , Jahanshahi M , Quinn N . Health‐related quality of life in patients with progressive supranuclear palsy. Mov Disord 2003;18(12):1464–1469. 10.1002/mds.10583. 14673883

[9] Cosentino G , Avenali M , Schindler A , et al. A multinational consensus on dysphagia in Parkinson's disease: screening, diagnosis and prognostic value. J Neurol 2022;269:1335–1352. 10.1007/s00415-021-10739-8. PMC8857094 34417870

[10] Schindler A , Pizzorni N , Cereda E , Cosentino G , Avenali M , Montomoli C , et al. Consensus on the treatment of dysphagia in Parkinson's disease. J Neurol Sci 2021;430. 10.1016/j.jns.2021.120008. 34624796

[11] Gandhi P , Steele CM . Effectiveness of interventions for dysphagia in Parkinson disease: a systematic review. Am J Speech Lang Pathol 2022;31:463–485. 10.1044/2021_AJSLP-21-00145. PMC9159671 34890260

[12] Menezes C , Melo A . Does levodopa improve swallowing dysfunction in Parkinson's disease patients? J Clin Pharm Ther 2009;34(6):673–676. 10.1111/j.1365-2710.2009.01031.x PMID: 20175800. 20175800

[13] Prosiegel M . Neurology of swallowing and dysphagia. Dysphagia. Heidelberg, Germany: Springer; 2017 Available from: https://link.springer.com/chapter/10.1007/174_2017_101.

[14] Varanese S , Di Ruscio P , Ben M Barek L , Thomas A , Onofrj M . Responsiveness of dysphagia to acute L‐dopa challenge in progressive supranuclear palsy. J Neurol 2014;261:441–442. 10.1007/s00415-013-7232-4, 24413640. 24413640

[15] Tomita S , Oeda T , Umemura A , et al. Impact of aspiration pneumonia on the clinical course of progressive supranuclear palsy: a retrospective cohort study. PLoS One 2015;10(8):e0135823. 10.1371/journal.pone.0135823. PMC4536232 26270456

[16] Wirth R , Dziewas R , Beck AM , Clavé P , Hamdy S , Heppner HJ , et al. Oropharyngeal dysphagia in older persons – from pathophysiology to adequate intervention: a review and summary of an international expert meeting. Clin Interv Aging 2016;11:189–208. 10.2147/CIA.S97481. PMC4770066 26966356

[17] Andersen PM , Abrahams S , Borasio GD , de Carvalho M , Chio A , Van Damme P , et al. EFNS guidelines on the clinical Management of Amyotrophic Lateral Sclerosis (MALS) ‐ revised report of an EFNS task force. Eur J Neurol 2012;19(3):360–375. 10.1111/j.1468-1331.2011.03501.x. 21914052

[18] Miller RG . The care of the patient with amyotrophic lateral sclerosis: drug, nutritional, and respiratory therapies. Neurology 2010;74(9):781. 10.1212/WNL.0b013e3181d38ced.

[19] Wirth R , Smoliner C , Jäger M , Warnecke T , Leischker AH , Dziewas R , et al. Guideline clinical nutrition in patients with stroke. Exp Transl Stroke Med 2013;5(1):1–11. 10.1186/2040-7378-5-14. PMC4176491 24289189

[20] Volkert D , Chourdakis M , Faxen‐Irving G , et al. ESPEN guidelines on nutrition in dementia. Clin Nutr 2015;34(6):1052–1073. 10.1016/j.clnu.2015.09.004 PMID: 26522922. 26522922

[21] Society AG . American Geriatrics Society feeding tubes in advanced dementia position statement. J Am Geriatr Soc 2014;62(8):1590–1593. 10.1111/jgs.12924. 25039796

[22] NICE . Dementia: Assessment, management and support for people living with dementia and their carers; 2018. (June 2018). Accessed online at: https://www.nice.org.uk/guidance/ng97. 30011160

[23] Sampson EL , Candy B , Jones L . Enteral tube feeding in older people with advanced dementia: findings from a Cochrane systematic review. Int J Palliat Nurs 2009;15(8):396–404. 10.12968/ijpn.2009.15.8.43799. 19773704

[24] Davies N , Barrado‐Martín Y , Vickerstaff V , et al. Enteral tube feeding for people with severe dementia. Cochrane Database Syst Rev 2021;2021(8). 10.1002/14651858.CD013503.pub2. PMC8407048 34387363

[25] Stall NM , Quinn KL , Van Der Steen JT , Trimble J , Clarfield AM , Mitchell SL . Enteral tube feeding in people with advanced dementia. BMJ 2025;389:389. 10.1136/bmj-2023-075326. 40374293

[26] Dommershuijsen LJ , Darweesh SKL , Ben‐Shlomo Y , Kluger BM , Bloem BR . The elephant in the room: critical reflections on mortality rates among individuals with Parkinson's disease. NPJ Parkinsons Dis Nature Research 2023;9:145. 10.1038/s41531-023-00588-9. PMC10587193 37857675

[27] Bäckström D , Granåsen G , Domellöf ME , Linder J , Mo SJ , Riklund K , et al. Early predictors of mortality in parkinsonism and Parkinson disease a population‐based study. Neurology 2018;91(22):E2045–E2056. 10.1212/WNL.0000000000006576. PMC6282235 30381367

[28] Respondek G , Stamelou M , Kurz C , et al. The phenotypic spectrum of progressive supranuclear palsy: a retrospective multicenter study of 100 definite cases. Mov Disord 2014;29(14):1758–1766. 10.1002/mds.26054. 25370486

[29] Pagano G , Ferrara N , Brooks DJ , Pavese N . Age at onset and Parkinson disease phenotype. Neurology 2016;86(15):1400–1407. 10.1212/WNL.0000000000002461. PMC4831034 26865518

[30] Macleod A , Henery R , Nwajiugo P , Scott N , Caslake R , Counsell C . Age‐related selection bias in Parkinson's disease research: are we recruiting the right participants? Parkinsonism Relat Disord 2018;55:128–133. 10.1016/j.parkreldis.2018.05.027. 29871791

[31] Goh YY , Saunders E , Pavey S , Rushton E , Quinn N , Houlden H , et al. Multiple system atrophy. Pract Neurol 2023;23:208–221. 10.1136/pn-2020-002797. PMC10314081 36927875

[32] Masrori P , Van Damme P . Amyotrophic lateral sclerosis: a clinical review. Eur J Neurol 2020;27:1918–1929. 10.1111/ene.14393. PMC7540334 32526057

[33] Ruoppolo G , Schettino I , Frasca V , et al. Dysphagia in amyotrophic lateral sclerosis: prevalence and clinical findings. Acta Neurol Scand 2013;128(6):397–401. 10.1111/ane.12136. 23668293

[34] Ishihara LS , Cheesbrough A , Brayne C , Schrag A . Estimated life expectancy of Parkinson's patients compared with the UK population. J Neurol Neurosurg Psychiatry 2007;78(12):1304–1309. 10.1136/jnnp.2006.100107. PMC2095626 17400591

[35] Testa D , Monza D , Ferrarini M , Soliveri P , Girotti F , Filippini G . Comparison of natural histories of progressive supranuclear palsy and multiple system atrophy. Neurol Sci 2001;22:247–251. 10.1007/s100720100021. 11731878

[36] Wenning GK , Ben Shlomo Y , Magalhaes M , Daniel SE , Quinn NP . Clinical features and natural history of multiple system atrophy: an analysis of 100 cases. Brain 1994;117:835−845. 10.1093/brain/117.4.835. 7922469

[37] Chiò A , Logroscino G , Hardiman O , et al. Prognostic factors in ALS: a critical review. Amyotroph Lateral Scler 2009;10:310–323. 10.3109/17482960802566824. PMC3515205 19922118

[38] Gallagher J , Gochanour C , Caspell‐Garcia C , et al. Long‐term dementia risk in Parkinson disease. Neurology 2024;103(5):e209699. 10.1212/WNL.0000000000209699. PMC11318527 39110916

[39] O'Sullivan SS , Massey LA , Williams DR , et al. Clinical outcomes of progressive supranuclear palsy and multiple system atrophy. Brain 2008;131(5):1362–1372. 10.1093/brain/awn065. 18385183

[40] Josephs KA , Dickson DW . Diagnostic accuracy of progressive supranuclear palsy in the Society for Progressive Supranuclear Palsy Brain Bank. Mov Disord 2003;18(9):1018–1026. 10.1002/mds.10488. 14502669

[41] Cui Y , Cao S , Li F , Feng T . Prevalence and clinical characteristics of dementia and cognitive impairment in multiple system atrophy: a systematic review and meta‐analysis. J Parkinsons Dis 2022;12(8):2383–2395. 10.3233/jpd-223444. 36336940

[42] Ringholz GM , Appel SH , Bradshaw M , Cooke NA , Mosnik DM , Schulz PE . Prevalence and patterns of cognitive impairment in sporadic ALS. Neurology 2005;65(4):586−590. 10.1212/01.wnl.0000172911.39167.b6. 16116120

[43] Murphy P , Albert SM , Weber CM , Del Bene ML , Rowland LP . Impact of spirituality and religiousness on outcomes in patients with ALS. Neurology 2000;55:1581–1584. 10.1212/wnl.55.10.1581. 11094123

[44] Martin NH , Landau S , Janssen A , et al. Psychological as well as illness factors influence acceptance of non‐invasive ventilation (NIV) and gastrostomy in amyotrophic lateral sclerosis (ALS): a prospective population study. Amyotroph Lateral Scler Frontotemporal Degener 2014;15(5–6):376–387. 10.3109/21678421.2014.886700 PMID: 24597488. 24597488

[45] Albert SM , Murphy PL , Del Bene ML , Rowland LP . Prospective study of palliative care in ALS: choice, timing, outcomes. J Neurol Sci 1999:169(1):108−113. 10.1016/S0022-510X(99)00227-0. 10540017

[46] Rowe JB , Holland N , Rittman T . Progressive supranuclear palsy: diagnosis and management. Practical Neurology. London, UK: BMJ Publishing Group; 2021:376–383. 10.1136/practneurol-2020-002794. PMC8461411 34215700

[47] Choo XY , Lim SY , Chinna K , et al. Understanding patients' and caregivers' perspectives and educational needs in Parkinson's disease: a multi‐ethnic Asian study. Neurol Sci 2020;41(10):2831–2842. 10.1007/s10072-020-04396-4. 32314118

[48] Kwak J , Wallendal M , Fritsch T , Leo G , Hyde T . Advance care planning and proxy decision making for patients with advanced Parkinson disease. South Med J 2014;107(3):178–185. 10.1097/smj.0000000000000075. 24937337

[49] Martin NH , Lawrence V , Murray J , et al. Decision making about gastrostomy and noninvasive ventilation in amyotrophic lateral sclerosis. Qual Health Res 2016;26(10):1366–1381. 10.1177/1049732315583661. 25918114

[50] Greenaway LP , Martin NH , Lawrence V , Janssen A , Al‐Chalabi A , Leigh PN , et al. Accepting or declining non‐invasive ventilation or gastrostomy in amyotrophic lateral sclerosis: patients' perspectives. J Neurol 2015;262(4):1002–1013. 10.1007/s00415-015-7665-z. 25683760

[51] Clarke G , Galbraith S , Woodward J , Holland A , Barclay S . Should they have a percutaneous endoscopic gastrostomy? The importance of assessing decision‐making capacity and the central role of a multidisciplinary team. Clin Med 2014;14(3):245–249. 10.7861/clinmedicine.14-3-245. PMC4952534 24889566

[52] Harwood RH . Feeding decisions in advanced dementia. J R Coll Physicians Edinb 2014;44(3):232–237. 10.4997/JRCPE.2014.310. 25318402

[53] Druml C , Ballmer PE , Druml W , et al. ESPEN guideline on ethical aspects of artificial nutrition and hydration. Clin Nutr 2016;35(3):545–556. 10.1016/j.clnu.2016.02.006. 26923519

[54] Brown L , Oswal M , Samra AD , et al. Mortality and institutionalization after percutaneous endoscopic gastrostomy in Parkinson's disease and related conditions. Mov Disord Clin Pract 2020;7(5):509–515. 10.1002/mdc3.12971. PMC7328413 32626795

[55] NICE . Parkinson's disease in adults; 2017. (July 2017). Accessed online at: https://www.nice.org.uk/guidance/NG71.

[56] Löser C , Aschl G , Hébuterne X , Mathus‐Vliegen EMH , Muscaritoli M , Niv Y , et al. ESPEN guidelines on artificial enteral nutrition ‐ Percutaneous endoscopic gastrostomy (PEG). Clin Nutr 2005;24:848–861. 10.1016/j.clnu.2005.06.013. 16261664

[57] Kobylecki C , Goh YY , Mohammad R , et al. Clinical practices and opinions toward gastrostomy use in patients with atypical parkinsonian syndromes: a National Survey in the UK. Mov Disord Clin Pract. 2024;11:1266–1273. 10.1002/mdc3.14196. PMC11489604 39189113

[58] Kobylecki C , Chelban V , Goh YY , et al. Frequency and outcomes of gastrostomy insertion in a longitudinal cohort study of atypical parkinsonism. Eur J Neurol 2024;31:e16258. 10.1111/ene.16258. PMID: 38407533. PMC11235814 38407533

[59] Mahale RR , Krishnan S , Divya KP , Jisha VT , Kishore A . Subtypes of PSP and prognosis: a retrospective analysis. Ann Indian Acad Neurol 2021;24(1):56–62. 10.4103/aian.AIAN_611_20. PMC8061531 33911380

[60] Nath U , Ben‐Shlomo Y , Thomson R , Lees A , Burn D . Clinical features and natural history of progressive supranuclear palsy ‐ a clinical cohort study. Neurology 2003;60(6):910–916. 10.1212/01.wnl.0000052991.70149.68. 12654952

[61] Sarkar P , Cole A , Scolding NJ , Rice CM . Percutaneous endoscopic gastrostomy tube insertion in neurodegenerative disease: a retrospective study and literature review. Clin Endosc 2017;50(3):270–278. 10.5946/ce.2016.106. PMC5475517 27737522

[62] Do HJ , Seo HG , Lee HH , Oh BM , Kim Y , Kim A , et al. Progression of oropharyngeal dysphagia in patients with multiple system atrophy. Dysphagia 2020;35(1):24–31. 10.1007/s00455-019-09990-z. 30852647

[63] Marois C , Amador MDM , Payan C , Lacomblez L , Bonnet AM , Degos B , et al. Outcome of gastrostomy in parkinsonism: a retrospective study. Parkinsonism Relat Disord 2017;43:110–113. 10.1016/j.parkreldis.2017.06.012. 28781200

[64] Homma T , Mochizuki Y , Tobisawa S , Komori T , Isozaki E . Cerebral white matter tau‐positive granular glial pathology as a characteristic pathological feature in long survivors of multiple system atrophy. J Neurol Sci 2020;416. 10.1016/j.jns.2020.117010. 32652361

[65] Lang A , Bardan E , Chowers Y , Sakhnini E , Fidder HH , Bar‐Meir S , Avidan B . Risk factors for mortality in patients undergoing percutaneous endoscopic gastrostomy. Endoscopy 2004;36(6):522–526. 10.1055/s-2004-814400 PMID: 15202049. 15202049

[66] Malmgren A , Hede GW , Karlström B , Cederholm T , Lundquist P , Wirén M , et al. Indications for percutaneous endoscopic gastrostomy and survival in old adults. Food Nutr Res 2011;55:6037. 10.3402/fnr.v55i0.6037. PMC3144742 21799666

[67] Kara O , Kizilarslanoglu MC , Canbaz B , et al. Survival after percutaneous endoscopic gastrostomy in older adults with neurologic disorders. Nutr Clin Pract 2016;31(6):799–804. 10.1177/0884533616648132. 27207937

[68] Wrigley S , Cullinane PW , Parmera JB , et al. Clinical diagnosis of progressive supranuclear palsy (PSP): a clinicopathological comparison of patients with confirmed PSP and clinical mimics. Mov Disord 2025;40:2116–2128. 10.1002/mds.30261. 40504076

[69] Tiankanon K , Aniwan S , Karuehardsuwan J , Wiangngoen S , Rerknimitr R . Factors affecting late complications of percutaneous endoscopic gastrostomy tube replacement. Clin Nutr ESPEN 2022;49:378–384. 10.1016/j.clnesp.2022.03.018. 35623840

[70] Goetz CG , Leurgans S , Lang AE , Litvan I . Progression of gait, speech and swallowing deficits in progressive supranuclear palsy. Neurology 2003;60(6):917–922. 10.1212/01.wnl.0000052686.97625.27. 12654953

[71] Yamazaki Y , Kobatake K , Hara M , Katagiri M , Matsumoto M . Nutritional support by “conventional” percutaneous endoscopic gastrostomy feeding may not result in weight gain in Parkinson's disease. J Neurol 2011;258:1561–1563. 10.1007/s00415-011-5971-7. 21547382

[72] Matino J . Feeding jejunostomy in patients with neurologic disorders. JAMA Surg 1981;116(2):169–171. 10.1001/archsurg.1981.01380140023005. 6810842

[73] Rimon E , Berner YN , Gindin J , Bass DD , Levy S . Low complication rate after insertion of percutaneous endoscopic gastrostomy by a geriatrics‐oriented team. J Am Geriatr Soc Blackwell Publishing Inc 1999;47(6):765–766. 10.1111/j.1532-5415.1999.tb01610.x. 10366186

[74] Heaney A , Tham T . Percutaneous endoscopic gastrostomies: attitudes of general practitioners and how management may be improved. Br J Gen Pract 2001;51:128–129. PMC1313928 11217626

[75] Khokhar N , Gill L . Percutaneous endoscopic gastrostomy: nine years experience in a tertiary care centre in Pakistan. J Pak Med Assoc 2005;55(3):108–110. 15852746

[76] Lee TH , Shih LN , Lin JT . Clinical experience of percutaneous endoscopic gastrostomy in Taiwanese patients—310 cases in 8 years. J Formos Med Assoc 2007;106(8):685–689. 10.1016/s0929-6646(08)60029-7. 17711805

[77] Cortés‐Flores AO , Álvarez‐Villaseñor A d S , Fuentes‐Orozco C , Ramírez‐Campos KM , Ramírez‐Arce A d R , Macías‐Amezcua MD , et al. Long‐term outcome after percutaneous endoscopic gastrostomy in geriatric Mexican patients. Geriatr Gerontol Int 2015;15(1):19–26. 10.1111/ggi.12215. 24372782

[78] Chang WK , Lin KT , Tsai CL , Chung CH , Chien WC , Lin CS . Trends regarding percutaneous endoscopic gastrostomy a nationwide population‐based study from 1997 to 2010. Medicine (United States) 2016;95(24):e3910. 10.1097/MD.0000000000003910. PMC4998481 27310995

[79] Barbosa M , Magalhaes J , Marinho C , Cotter J . Predictive factors of early mortality after percutaneous endoscopic gastrostomy placement: the importance of C‐reactive protein. Clin Nutr ESPEN 2016;14:19–23. 10.1016/j.clnesp.2016.04.029. 28531394

[80] Lee J , Shim KN , Lee KH , et al. Clinical course of percutaneous endoscopic gastrostomy: a single‐center observational study. Korean J Gastroenterol 2018;71(1):24–30. 10.4166/kjg.2018.71.1.24. 29361810

[81] Pih GY , Na HK , Ahn JY , et al. Risk factors for complications and mortality of percutaneous endoscopic gastrostomy insertion. BMC Gastroenterol 2018;18(1):101. 10.1186/s12876-018-0825-8. PMC6025834 29954339

[82] Park SK , Kim JY , Koh SJ , Lee YJ , Jang HJ , Park SJ . Complications of percutaneous endoscopic and radiologic gastrostomy tube insertion: a KASID (Korean Association for the Study of intestinal diseases) study. Surg Endosc 2019;33(3):750–756. 10.1007/s00464-018-6339-1. 30132209

[83] Duzenli T , Ketenci M , Akyol T , Koseoglu H , Tanoglu A , Kaplan M , Yazgan Y . Predictive factors of complications and 30‐day mortality in patients undergoing percutaneous endoscopic gastrostomy: the utility of c‐reactive protein to albumin ratio. Acta Gastroenterol Belg 2021;84(2):283–288. 10.51821/84.2.283. 34217176

[84] Akkuzu MZ , Sezgin O , Ucbilek E , et al. Efficacy and safety of percutaneous endoscopic gastrostomy in elderly patients aged over 65: a tertiary center long‐term results. Med Bull Haseki 2021;59(2):128–132. 10.4274/haseki.galenos.2021.6429.

[85] Zalar A , Guédon C , Basso A , Ducrotté P . Percutaneous endoscopic gastrostomy in patients with neurological swallowing disorders. Results of an international multicentric prospective study. Acta Gastroenterol Latinoam 2004;34(3):127–132. 15742927

[86] Tudor C , Branescu C , Savlovschi C , El‐Khatob A , Pantu H , Nica A , et al. Gastrostomy with peritoneal collar versus percutaneous endoscopic gastrostomy. J Med Life 2016;9:408–412. 10.22336/jml.2016.0415. PMC5141402 27928446

[87] Tomasello G , Bellavia M , Damiano G , Palumbo VD , Spinelli G , Cacciabaudo F , et al. Enteral nutrition: our experience with percutaneous endoscopic gastrostomy (PEG) and revision of literature. Prog Nutr 2012;14(2):141–143.

[88] Gençosmanoglu R , Şad O , Avsar E , Hamzaoglu H , Ozdogan O , Kalayci C , et al. Percutaneous endoscopic gastrostomy: results of 50 cases. Marmara Med J 2000;13(4):212–218.

[89] Luman W , Kwek KR , Loi KL , Chiam MA , Cheung WK , Ng HS , et al. Percutaneous endoscopic gastrostomy‐indications and outcome of our experience at the Singapore General Hospital. Singapore Med J 2001;42(10):460–465. 11874149

[90] Bine JE , Frank EM , Mcdade HL . Dysphagia and dementia in subjects with Parkinson's disease. Dysphagia 1995;10:160–164. 10.1007/BF00260970. 7614855

[91] Goy ER , Carter JH , Ganzini L . Parkinson disease at the end of life: caregiver perspectives. Neurology 2007;69(6):611–612. 10.1212/01.wnl.0000266665.82754.61. 17679683

[92] Sato T , Shiobara M , Nishizawa M , Shimohata T . Nutritional status and changes in body weight in patients with multiple system atrophy. Eur Neurol 2017;77(1–2):41–44. 10.1159/000453395. 27894118

[93] Tye CB , Gardner PA , Dion GR , Simpson CB , Dominguez LM . Impact of fiberoptic endoscopic evaluation of swallowing outcomes and dysphagia Management in Neurodegenerative Diseases. Laryngoscope 2021;131(4):726–730. 10.1002/lary.28791. 32542698

[94] El Fassi N , Gallois Y , Crestani S , Fichaux‐Bourrin P , Ory F , Fabbri M , et al. Pharyngolaryngeal semiology and prognostic factors in multiple system atrophy. Eur Arch Otorhinolaryngol 2022;279(9):4473–4483. 10.1007/s00405-022-07410-x. PMC9363394 35513505

[95] Kim DS , Jones RN , Shireman TI , Kluger BM , Friedman JH , Akbar U . Trends and outcomes associated with gastrostomy tube placement in common neurodegenerative disorders. Clinical Parkinsonism & Related Disorders 2021;4. 10.1016/j.prdoa.2020.100088. PMC8299983 34316666

[96] Payne C , Wiffen PJ , Martin S . Interventions for fatigue and weight loss in adults with advanced progressive illness. Cochrane Database Syst Rev 2012. 10.1002/14651858.CD008427.pub2. 22258985

[97] Rittman T , Coyle‐Gilchrist IT , Rowe JB . Managing cognition in progressive supranuclear palsy. Neurodegenerative disease management London: Future Science Group; 2016:499–508. 10.2217/nmt-2016-0027. PMC5134756 27879155

[98] Miyasaki JM . Treatment of advanced Parkinson disease and related disorders. Continuum (Minneap Minn) 2016;22(4):1104–1116. 10.1212/CON.0000000000000347 27495200

[99] Calandra‐Buonaura G , Alfonsi E , Vignatelli L , Benarroch EE , Giannini G , Iranzo A , et al. Dysphagia in multiple system atrophy consensus statement on diagnosis, prognosis and treatment. Parkinsonism Relat Disord 2021;86:124–132. 10.1016/j.parkreldis.2021.03.027. 33839029

[100] Stavroulakis T , McDermott CJ . Enteral feeding in neurological disorders. Practical Neurology. London UK: BMJ Publishing Group; 2016:352–361. 10.1136/practneurol-2016-001408. 27152026

[101] Barichella M , Cereda E , Pezzoli G . Major nutritional issues in the management of Parkinson's disease. Mov Disord 2009;24:1881–1892. 10.1002/mds.22705. PMID: 19691125. 19691125

[102] Olanow C , Watts R , Koller W . An algorithm (decision tree) for the management of Parkinson's disease (2001): treatment guidelines. Neurology 2001;56(5):S1–S88. 10.1212/wnl.56.suppl_5.s1. 11402154

[103] Lewis SJ , Gangadharan S , Padmakumar CP . Parkinson's disease in the older patient. Clin Med (Lond) 2016;16:376–384. 10.7861/clinmedicine.16-4-376. PMC6280201 27481385

[104] Walker RW . Palliative care and end‐of‐life planning in Parkinson's disease. J Neural Transm 2013;120:635–638. 10.1007/s00702-013-0967-3. 23328948

[105] Fanciulli A , Wenning GK . Multiple‐system atrophy. N Engl J Med 2015;372(3):249–263. 10.1056/NEJMra1311488. 25587949

[106] Gilbert R , Khemani P . Treatment of advanced Parkinson's disease. J Geriatr Psychiatry Neurol 2022;35(1):12–23. 10.1177/0891988720988904. 33511915

[107] Warnecke T , Dziewas R . Swallowing in progressive supranuclear palsy and implications for nutrition. Diet and Nutrition in Dementia and Cognitive Decline. Amsterdam, Netherlands: Elsevier Inc.; 2015:1135–1141. 10.1016/B978-0-12-407824-6.00106-3.

[108] Evans S , Soar N , Lang A , Sommerville P , Archer S , Birns J . Risk feeding in the advanced stages of Parkinson's disease. Prog Neurol Psychiatry 2019;23(4):15–18. 10.1002/pnp.549.

[109] Kim DS , Kunicki ZJ , Philips OW , Jones RN , Friedman JH , Kluger B , Akbar U . Racial and geographic disparities with gastrostomy tube placement in dementia and parkinsonian disorders. Parkinsonism Relat Disord 2021;91:28–31. 10.1016/j.parkreldis.2021.08.016. PMC9486970 34479055

[110] Baijens LWJ , Clavé P , Cras P , Ekberg O , Forster A , Kolb GF , et al. European society for swallowing disorders ‐ European union geriatric medicine society white paper: oropharyngeal dysphagia as a geriatric syndrome. Clin Interv Aging 2016;2016(11):1403–1428. 10.2147/CIA.S107750. PMC5063605 27785002

[111] Burgos R , Bretón I , Cereda E , et al. ESPEN guideline clinical nutrition in neurology. Clin Nutr 2018;37(1):354–396. 10.1016/j.clnu.2017.09.003. 29274834

[112] Coon EA , Golden EP , Bryarly M , McGregor T , Nguyen BN , Moutvic MA , et al. A call for multiple system atrophy centers of excellence. Clin Auton Res 2022;32:205–208. 10.1007/s10286-022-00866-1. 35552950

[113] Russ KB , Phillips MC , Wilcox CM , Peter S . Percutaneous endoscopic gastrostomy in amyotrophic lateral sclerosis. Am J Med Sci 2015;350(2):95–97. 10.1097/MAJ.0000000000000517. 26135224

[114] Dorst J , Dupuis L , Petri S , et al. Percutaneous endoscopic gastrostomy in amyotrophic lateral sclerosis: a prospective observational study. J Neurol 2015;262(4):849–858. 10.1007/s00415-015-7646-2. 25618254

[115] Meier DE , Ahronheim JC , Morris J , Baskin‐Lyons S , Morrison RS . High short‐term mortality in hospitalized patients with advanced dementia lack of benefit of tube feeding. Arch Intern Med 2001;161:594–599. 10.1001/archinte.161.4.594. 11252121

[116] Ticinesi A , Nouvenne A , Lauretani F , Prati B , Cerundolo N , Maggio M , Meschi T . Survival in older adults with dementia and eating problems: to PEG or not to PEG? Clin Nutr 2016;35(6):1512–1516. 10.1016/j.clnu.2016.04.001. 27091773

[117] Murphy LM , Lipman TO . Percutaneous endoscopic gastrostomy does not prolong survival in patients with dementia. Arch Intern Med 2003;163:1351–1353. 10.1001/archinte.163.11.1351. 12796072

[118] Pedrosa AJ , Feldmann S , Klippel J , Volberg C , Weck C , Lorenzl S , et al. Factors associated with preferred place of care and death in patients with Parkinson's disease: a cross‐sectional study. J Parkinsons Dis 2024;14(3):589–599. 10.3233/JPD-230311. PMC11091558 38457148

[119] Spataro R , Ficano L , Piccoli F , La Bella V . Percutaneous endoscopic gastrostomy in amyotrophic lateral sclerosis: effect on survival. J Neurol Sci 2011;304(1–2):44–48. 10.1016/j.jns.2011.02.016. 21371720

[120] Sulistyo A , Abrahao A , Freitas ME , Ritsma B , Zinman L . Enteral tube feeding for amyotrophic lateral sclerosis/motor neuron disease. Cochrane Database Syst Rev 2023. 10.1002/14651858.CD004030.pub4. PMC10413437 37579081

