# Supplementary material for: Enteral Nutrition in Idiopathic Parkinson's Disease and Atypical Parkinsonism: A Systematic Review
Source: Mov Disord Clin Pract. 2026 May 1:10.1002/mdc3.70653. Online ahead of print. doi: 10.1002/mdc3.70653 (PMC13339564; doi:10.1002/mdc3.70653)
Supplement: Supplementary file 1 — Table S1. Frequency of parkinsonism as indication for PEG insertion in unselected/general endoscopy lists (n = 21) Table S2. Prevalence of enteral feeding in parkinsonism groups (n = 9) Table S3. Summary of recommendations regarding enteral nutrition in PD and atypical parkinsonism (n = 22) Table S4. Detailed mortality and morbidity data after enteral feeding initiation (n = 14) Table S5. Detailed recommendations on PEG placement in parkinsonism; ordered according to overall view towards PEG placement (n = 22) Table S6. Risk of bias assessment for studies with a comparator group, using The Risk Of Bias In Non‐randomized Studies – of Interventions, Version 2 (ROBINS‐I V2) assessment tool (n = 2) Table S7. Risk of bias assessment for studies without a comparator group, using the JBI Cohort Study checklist (n = 14) [file MDC3-9999-0-s001.docx]

Supplementary information

**Supplementary Table 1. Frequency of parkinsonism as indication for PEG insertion in unselected/general endoscopy lists (n = 21)**

| **Year** | **Author** | **Country** | **Gastrostomy types** | **Total no. of gastrostomies** | **Disease group** | **No. in disease group** | **% in disease group** | **Comment** |
| --- | --- | --- | --- | --- | --- | --- | --- | --- |
| 1981 | Matino | USA | Feeding jejunostomy | 44 | PD | 4 | 9.1 |  |
| 1999 | Rimon et al | Israel | PEG | 214 | PD | 15 | 7.0 |  |
| 2000 | Gencosmanoglu et al | Turkey | PEG | 50 | PD | 2 | 4.0 |  |
| 2001 | Luman et al | Singapore | PEG | 174 | PD | 8 | 4.6 |  |
| 2001 | Heaney | Northern Ireland | PEG | 275 | PD | n/a | 3.0 |  |
| 2004 | Zalar | Spain | PEG | 109 | PD | 3 | 2.8 | 11 deaths total, 10/11 were in ‘neurological disorder’ group. Mean follow-up 3 months |
| 2004 | Lang et al | Israel | PEG | 502 | PD | 28 | 5.0 | 2 died within 30 days, no association with indication |
| 2005 | Khokar | Pakistan | PEG | 182 | PD | 5 | 2.7 |  |
| 2007 | Lee et al | Taiwan | PEG | 308 | Parkinsonism | 21 | 6.8 |  |
| 2011 | Malmgren et al | Sweden | PEG | 201 | PD | 12 | 6.0 | 92% done for dysphagia, 8% for 'inability to eat' |
| 2012 | Tomasello | Italy | PEG | 44 | PD | n/a | 37.0 |  |
| 2015 | Cortes-Flores et al | Mexico | PEG in >70 year-olds | 110 | PD | 12 | 9.0 | Serum albumin and co-morbidities associated with early and late mortality following PEG |
| 2016 | Tudor et al | Romania | PEG + GPC | 94 | PD | 5 | 5.3 |  |
| 2016 | Chang et al | Taiwan | PEG | 5,532  (1,923 in 1997-2004 and 3,609 in 2005-2010) | PD | 168  (59 in 1997-2004 and 109 in 2005-2010) | 3.0 | Rising no. of PEGs over time, rise in mortality, rise in 'other' indication and complexity of medical morbidities |
| 2016 | Barbosa | Portugal | PEG | 135 | PD | 7 | 5.2 | ‘Neurological’ indications had better outcomes than ‘Malignancy’ |
| 2016 | Kara et al | Turkey | PEG | 500 | PD | n/a | 5.8 |  |
| 2018 | Lee et al | Korea | PEG | 137 | PD | 10 | 7.3 | No acute/chronic outcome differences between those <65 and >65 |
| 2018 | Pih et al | Korea | PEG | 401 | PD | 61 | 15.2 |  |
| 2019 | Park et al | Korea | PEG + PRG | 418 | PD | 44 | 10.5 | PEG preferred in ‘neurological conditions’, as greater overall risk but highest with PRG |
| 2021 | Duzenli et al | Turkey | PEG | 253 | Advanced Parkinsonism | 12 | 4.7 |  |
| 2021 | Akkuzu | Turkey | PEG | 140 | PD | 6 | 4.3 |  |
|  |  |  |  |  |  | **Weighted average (%)** | 5.1 |  |

*GPC – gastrostomy with peritoneal collar; PEG- percutaneous endoscopic Gastrostomy; PRG - percutaneous radiologic gastrostomy*

**Supplementary Table 2. Prevalence of enteral feeding in parkinsonism groups (n = 9)**

| **Year** | **Author** | **Country** | **Population** | **Study type** | **Study period** | **Findings** |
| --- | --- | --- | --- | --- | --- | --- |
| 1995 | Bine et al | USA | 19 PD | Retrospective, cohort | 1985-1990 | PEG incidence 42% in PD+dementia group and <5% in PD without dementia |
| 2007 | Goy et al | USA | 47 PD | Caregiver interviews | 6-53 months after patient death | 26% 'fed by tube' (including NG) in last month of life |
| 2017 | Sato et al | Japan | 130 MSA | Prospective, cohort | 2001-2014 | 9% 'tube feeding'; no difference between MSA-C + MSA-P |
| 2020 | Homma et al | Japan | 14 MSA | Retrospective | 2000-2014 | 64% ‘tube fed’ |
| 2020 | Do et al | Korea | 59 MSA | Retrospective+/- telephone interview | 1999-2017 | 25 (42.4%) had '*tube feeding*', median latency 7 years from onset of MSA symptoms. 12 deaths total, 7 dependent on feed at time of death |
| 2020 | Tye et al | USA | 209 parkinsonism: 26% PD, 5.1% PSP, 32% ALS | Retrospective | 2008-2019 | Patients reviewed at Fiberoptic endoscopic evaluation of swallowing (FEES) assessments. 10% PD (vs 40% ALS) referred for PEG. PD *‘more likely to benefit from swallow therapy’* vs ALS |
| 2021 | Mahale et al | India | 334 PSP | Retrospective | 1996-2017 | 34 (10.1%) required PEG, mean 5.6 years from disease onset. Longer disease duration to PEG in PSP-P (vs PSP‑RS/-CBS/‑F/-PGF) |
| 2021a | Kim et al | USA | 78,562 PD | Retrospective | 1990-2010 | PEG insertion rate fell by average 3.4%/year across study period. Aggregated 5-year results (per 1,000 PD cases): 19.7 (1990-1994) to 13.8 (2005-2009). Greater length of hospital stay and rates of long-term facility living in those with PEG than those without |
| 2022 | El Fassi et al | France | 101 MSA | Retrospective | n/a | 22% received enteral feeding (NG/gastrostomy), 6% refused; no relationship between PEG and MSA phenotype |

**Supplementary Table 3. Summary of recommendations regarding enteral nutrition in PD and atypical parkinsonism (n = 22)**

| **Year** | **Author** | **Country** | **Study/Article type** |
| --- | --- | --- | --- |
| 1. **Highlights lack of evidence and/or need for ‘individualised’ approach** | | | |
| 2005 | Löser et al | Europe  *ESPEN* | Consensus  *Artificial enteral nutrition* |
| 2012 | Payne et al | UK | Cochrane Review  *Fatigue and weightloss in progressive illness* |
| 2016 | Wirth et al | International | Review  *Dysphagia in older people* |
| 2016 | Rittman et al | UK | Review  *Cognition in PSP* |
| 2016 | Miyasaki | USA | Review  *Treatment of advanced PD* |
| 2020 | Umemoto et al | Japan | Review  *Dysphagia in PD/PD+* |
| 2021 | Schindler et al | International  *Multinational Consensus Conference* | Consensus  *Dysphagia in PD* |
| 2021 | Calandara-Buonaura et al | Europe  *International Consensus Conference* | Consensus  *Dysphagia in MSA* |
| 2016 | Stavroulakis & McDermott | UK | Review  *Enteral feeding in neurological conditions* |
| 1. **Enteral feeding as ‘last resort’** | | | |
| 2001 | Olanow et al | USA | Guidelines  *Management of PD* |
| 2009 | Barichella et al | Italy | Review  *Nutrition in PD* |
| 1. **Enteral feeding as only suitable for a minority of patients** | | | |
| 2016 | Lewis et al | Australia | Opinion  *PD in older people* |
| 1. **Highlight importance of ACP in enteral feeding decision-making** | | | |
| 2013 | Walker | UK | Review  *Palliative care in PD* |
| 2015 | Fanciulli & Wenning | Austria/Italy | Review  *MSA* |
| 2022 | Gilbert et al | USA | Review  *Advanced PD* |
| 1. **Enteral feeding should be considered (in advanced PSP)** | | | |
| 2015 | Warnecke & Dziewas | Germany | Book chapter  *Swallowing and nutrition in PSP* |
| 1. **Highlight lack of efficacy (e.g. on aspiration pneumonia, quality of life) of enteral feeding in parkinsonism** | | | |
| 2019 | Evans et al | UK | Original research |
| 1. **Avoid enteral feeding; equate advanced PD and dementia, given high dementia prevalence** | | | |
| 2021a | Kim et al | USA | Original research |
| 2021b | Kim et al | USA | Original research |
| 1. **Lack of specific guidance for parkinsonism** | | | |
| 2016 | Baijens et al | Europe  *European Society for Swallowing Disorders- EU Geriatric Medicine Society* | Position paper  *Dysphagia in geriatrics* |
| 2018 | Burgos et al | Europe  *ESPEN* | Guideline  *Nutrition in Neurology* |
| 2022 | Coon et al | USA | Letter  *MSA Centres of Excellence* |

**Supplementary Table 4. Detailed mortality and morbidity data after enteral feeding initiation (n=14)**

| **Year** | **Author** | **Country** | **Population** | **Study period** | **Findings** |
| --- | --- | --- | --- | --- | --- |
| 2003 | Goetz et al | USA | 50 PSP | 1989-2000 | Feeding tube (NG/PEG) was first milestone in 8% (median 87 months from symptom onset); infrequent compared to speech (55%) and gait (48%) deficits.  54% needed tube during study (mean 11.5 months follow-up) |
| 2003 | Nath et al | UK | 187 PSP | 1999-2001 | Early PEG insertion trended towards higher mortality in PSP (*HR 1.97, p 0.10*).  9% PEG insertion prevalence; average 5-year latency from disease onset to PEG. |
| 2004 | Lang et al | Israel | 28 PD with PEG | 1995-2001 | PD not significantly associated with mortality or survival compared with other PEG indications on univariate analysis; 7% (2) died within 30 days |
| 2011 | Yamazaki et al | Japan | 8 PD with PEG | 1996-2009 | *‘‘Conventional’’ PEG feeding may not result in weight gain in PD*  Study acknowledges contradictory findings from other studies, including gender differences |
| 2011 | Malmgren et al | Sweden | 11 PD with PEG (> 65 years old) | 1997-2000 | PD median survival (233 days) was the longest in the study group; no statistics performed, overall median survival was ~120 days.  PD % mortality at 30 days (9), 3 months (18), 6 months (36) and 12 months (55). |
| 2015 | Tomita et al | Japan | 90 PSP | 2006-2014 | 24% of total PSP group developed pneumonia; 91% of these received non-oral feeding, whilst 20% received PEG.  Of 16 who died, 10 had PEG.  Ave. time from aspiration to PEG placement was *‘a few months’* |
| 2016 | Goh et al | Singapore | 194 PD | 2010-2014 | Higher mortality in those who received (HR 2.29) and those who rejected (HR 4.61) enteral feeding compared to those who had oral feeding (adjusted for confounders). More admissions for pneumonia in those who received (61.8%) and those who rejected (66.7%) enteral feeding than those who had oral feeding (38.8%) without dysphagia. |
| 2016 | Kara et al | Turkey | 29 PD with PEG (> 60 years old) | 2005-2015 | PD associated with increased mortality risk in univariate but not in multivariate analysis (stroke significant).  2- year PD+PEG mortality rate: 72%  Overall ‘neurological’ group data (6% PD): median survival - 13.9 months, 30-day mortality rate - 11.3%, 1-year mortality rate - 46.8%. |
| 2017 | Sarkar et al | UK | 13 PD/PD+ with PEG | 2009-2013 | PD, MSA, PSP - 32% of total group. Total group 30-day mortality - 8%; complication rate - 87.5%  PEG had ‘h*igher perioperational distress* vs RIG/PIG; no difference in survival or complications  20% malnourished pre-procedure (defined as albumin <30 g/L) |
| 2017 | Marois et al | France | 32 parkinsonism with PEG (7 PD, 15 MSA, 5 PSP, 3 CBD, 2 DLB) | 2008-2014 | Mortality rate: 59.4% - one year; 65.6% - two years  At least one adverse event – 69%  Median post-PEG survival - 186 days  Total dependency before gastrostomy associated with worse survival. |
| 2018 | Pih et al | Korea | 61 PD with PEG | 2005-2015 | ‘Other’ neurologic group (including PD) had a significantly lower incidence of 30-day mortality than stroke and malignancy groups.  30-day mortality rate 5.0% across all disease groups. |
| 2020 | Brown et al | UK | 93 parkinsonism with PEG  (58 PD/10 PSP/5 MSA/3 LBD/7 vascular) | 2005-2017 | 7-day mortality rate - 0%, 30-day mortality - 6.0%.  Median survival 422 days [366 days (2005-2010), 532 days (2011-2017)]  47% readmitted in the year after PEG insertion. 32% admitted from home were institutionalised on discharge after PEG insertion.  Prior to PEG, 23% had ACP in place, 83% had documented risk/benefit discussion.  ‘*Median survival in PD dementia - 344 days (584 days if those taking rivastigmine are reclassified as dementia)- therefore,* may be not futile |
| 2020 | Homma et al | Japan | 14 MSA | 2000-2014 | 7 (50%) had both tube feeding and tracheotomy; 71.4% of them were alive > 10 years after disease onset |
| 2022 | Tiankanon et al | Thailand | 42 PD with PEG | 2015-2020 | 33% had PEG-related complications > 6 months after procedure (e.g. infection); comparable to other diseases. |

**Supplementary Table 5. Detailed recommendations on PEG placement in parkinsonism; ordered according to overall view towards PEG placement (n = 22)**

| **Year** | **Author** | **Country** | **Study/Article type** | **Position** |
| --- | --- | --- | --- | --- |
| **Highlights lack of evidence and/or need for ‘individualised’ approach** | | | | |
| 2005 | Löser et al | Europe  *ESPEN* | Consensus  *Artificial enteral nutrition* | ‘*Neurological disorders’* inc. PD listed as indication. *‘The individual benefits with regard to quality of life is expected to be lower in older patients and patients with complex and severe co-morbidity and therefore individual indications…be considered more critically in these patients’* |
| 2012 | Payne et al | UK | Cochrane Review  *Fatigue and weightloss in progressive illness* | *‘Lack of robust data on interventions [*Includes gastrostomy] *to manage fatigue and/or unintentional weight loss in the advanced stage of progressive illnesses such as…Parkinson’s Disease’*  No specific studies included for PD |
| 2016 | Wirth et al | International | Review  *Dysphagia in older people* | *‘Malnutrition and aspiration pneumonia are highly prevalent in this disease and some of these patients with advanced disease may need tube feeding. However, the indication and effects of tube feeding in this disease are not yet studied. The most common indication for the insertion of a feeding tube seems to be primarily the safe and time-controlled provision of medication…the tube can also be used for nutrition’* |
| 2016 | Rittman et al | UK | Review  *Cognition in PSP* | ‘L*ack of evidence to help with the timing of PEG placement’*  References MND study as possibly relevant to PEG timing in PSP |
| 2016 | Miyasaki | USA | Review  *Treatment of advanced PD* | *‘There is no evidence that PEG feeding in PD improves mortality or improves quality of life. In selected cases, PEG for nutrition or medication administration may be effective; however, discussing the possibility of discontinuing enteral feeding may be helpful prior to placing the PEG tube’.* **No supporting references** |
| 2020 | Umemoto et al | Japan | Review  *Dysphagia in PD/PD+* | *‘PSP patients ultimately require PEG feeding within a few months after the initial development of pneumonia. Nevertheless, whether or not PEG placement prolongs the survival time (actuarially corrected to 6-10 years) is unclear… If the risk of aspiration increases and the management of oral nutrition is difficult, nutrition combined with NGT or PEG feeding should be considered. The establishment of a guideline for the treatment and rehabilitation of dysphagia in PD and PRD is expected’* |
| 2021 | Schindler et al | International  *Multinational Consensus Conference* | Consensus  *Dysphagia in PD* | *‘There are no PD-specific recommended criteria to guide PEG placement in daily practice and there are no data indicating that tube feeding prolongs survival in PD or improves QOL. Accordingly, we suggest referring to available international guidelines dealing with this issue* [i.e. ESPEN]*’*  *‘PEG should be placed in case of inadequate oral intake expected to be longer than 4 weeks resulting in involuntary body weight loss (≥5% in 1*  *month or ≥ 10% in 3 months) and/or significant risk of prandial aspiration exceeding the risk of aspiration of reflux…PEG feeding should be carefully considered on an individual basis taking into account patient and family choice, caregiving context, health ethics, prognosis and QOL…In case of dementia, PEG insertion is not indicated’* |
| 2021 | Calandara-Buonaura et al | Europe  *International*  *Consensus Conference* | Consensus  *Dysphagia in MSA* | *‘PEG feeding may be applied when there is a severe risk of malnutrition, dehydration, and pulmonary complications. There is no evidence that PEG improves survival or quality of life in MSA’*  *‘Studies to determine at which disease stage PEG placement is appropriate are warranted in order to guide physicians. Finally, it is*  *unknown whether this nutritional modality has different effects on*  *prognosis in MSA-P and MSA-C. By combining instrumental methods*  *with reliable rating scales, it might prove possible to identify the most*  *appropriate time (if any) for PEG placement, establishing factors on*  *which to base this decision, as well as the impact of PEG on survival and*  *quality of life’* |
| 2016 | Stavroulakis & McDermott | UK | Review  *Enteral feeding in neurological conditions* | Enteral feeding ‘*recommended’* in PD  *‘Currently, there is no evidence on the effectiveness of interventions, such as enteral feeding, on the improvement of the nutritional status, quality of life or survival of patients with Parkinson’s disease. Guidance from the National Collaborating Centre for Chronic Conditions (NCC-CC), on behalf of NICE, recommends an individual approach for the management of problems associated with eating and swallowing in patients with Parkinson’s disease, with the aim to anticipate and prevent complications where possible…* *gastrostomy feeding can be used for the*  *long-term nutritional support of patients with PD’* |
| **PEG as ‘last resort’** | | | | |
| 2001 | Olanow et al | USA | Guidelines  *Management of PD* | *‘Invasive interventions, such as a feeding gastrostomy, are a last resort’* |
| 2009 | Barichella et al | Italy | Review  *Nutrition in PD* | PEG included as last option in treatment algorithm for PD nutrition; no relevant references regarding this |
| **PEG as only suitable for a minority of patients** | | | | |
| 2016 | Lewis et al |  |  | *‘In a very small number of cases, consideration of enteral feeding via a percutaneous endoscopic gastrostomy tube is warranted’*  **No supporting references** |
| **Highlight importance of ACP in PEG decision-making** | | | | |
| 2013 | Walker | UK | Review  *Palliative care in PD* | *‘ACP in PD should be encouraged in relation to interventions such as PEG tubes...Practice in relation to this* [PEG in PD] *is variable, and the evidence base is limited. In the UK PEG tubes are rarely used in PD patients’* |
| 2015 | Fanciulli & Wenning | Austria/Italy | Review  *MSA* | *‘In advanced stages* [of MSA]*, a PEG allows for enteral feeding and lowers the risk of aspiration pneumonia, but it needs to be discussed with the patient well in advance in order to obtain informed consent’*  **No supporting references** |
| 2022 | Gilbert et al | USA | Review  *Advanced PD* | *‘An Advanced Directive [AD] should be in place to guide providers on invasive feeding measures such as inserting a feeding tube … If an AD is not available and the patient is mentally impaired, the patient’s legal and medical decision-maker should be consulted on invasive measures of feeding…it does not prevent aspiration pneumonia. It may potentially set up the patient for complications… There is a paucity of literature on the improvement of quality of life with gastrostomy tubes in PD. Therefore, the decision to insert a feeding tube should be made in an informed manner after the pros and cons are fully considered’* |
| **Positive view towards PEG in parkinsonism (+/- specific timing guidance)** | | | | |
| 2015 | Warnecke & Dziewas | Germany | Book chapter  *Swallowing and nutrition in PSP* | *‘A PEG should be considered when a PSP patient cannot meet his or her nutritional needs by oral food intake (unjustified weight loss of more than 10%), and/or when there is endoscopic or videofluoroscopic evidence that all types of textures are aspirated without any improvement by nonpharmacological therapeutic interventions and dopaminergic medication, and/or when it takes longer than 1 h to feed the patient* (Ref)*’* |
| **Negative view towards PEG in parkinsonism** | | | | |
| 2019 | Evans et al | UK | Original research | *‘Enteral feeding via gastrostomy may provide a longer term modality of nutritional support but may impact negatively on comfort, dignity and*  *quality of life* **[no supporting references]***…does not prevent aspiration pneumonia in patients with PD and has been shown to have a median survival post-procedure of 186 (62–309) days…with level of dependency being significantly negatively associated with survival. Hence, in advanced stages of PD, often with severe dementia or towards end of life, the multidisciplinary team is prompted to consider other options’* |
| 2021a | Kim et al | USA | Original research | *‘While there are no controlled trials, observational data in PD suggest no survival benefit (Ref). As the vast majority of advanced PD patients develop dementia, they may be treated under this general guideline (i.e. against GT placement)…* *We believe that appropriate expectation and discussion between physicians, patients/caregivers addressing the goals of care may help inform the post‐GT trajectory, and potentially decrease the likelihood of unnecessary or undesired nursing home placement’* |
| 2021b | Kim et al | USA | Original research | *‘Given the high prevalence of dementia in advanced stages of PD – a time when GT placement may be considered – one can carefully extrapolate from the AGS* [American Geriatric Society] *recommendation to avoid this procedure, albeit individual cases may not fall into this generalisation’* |
| **Lack of PEG specific guidance for parkinsonism** | | | | |
| 2016 | Baijens et al | Europe  *European Society for Swallowing Disorders- EU Geriatric Medicine Society* | Position paper  *Dysphagia in geriatrics* | *‘PEG … is recommended in patients who need long-term EN (4–6 weeks) such as those with chronic or progressive diseases…* *controversial discussion over whether PEG can prevent aspiration pneumonia’.* Explores ethical, legal, economic and medical aspects of feeding decisions in geriatric population; no specific recommendations for PD |
| 2018 | Burgos et al | Europe  *ESPEN* | Guideline  *Nutrition in Neurology* | *‘PD patients should receive medical nutrition therapy to improve well-being and quality of life. Medical nutrition therapy should be tailored to individual requirements’*  No specific comment on PEG in PD; by comparison, guidance is included for other disease groups (e.g. ALS) |
| 2022 | Coon et al | USA | Letter  *MSA Centres of Excellence* | No comment on PEG decision-making in MSA, only that *‘nutritionists guide tube feed recommendations’* |

**Supplementary Table 6. Risk of bias assessment for studies with a comparator group, using The Risk Of Bias In Non-randomized Studies – of Interventions, Version 2 (ROBINS-I V2) assessment tool (n = 2)**

| **Author Year** | **Study Design** | **Item 1** | **Item 2** | **Item 3** | **Item 4** | **Item 5** | **Overall** |
| --- | --- | --- | --- | --- | --- | --- | --- |
| **Goh 2016** | Retrospective; Cohort with comparator | Some Concern | High | Some Concern | Low | Low | High |
| **Kobylecki 2024** | Retrospective; Cohort with comparator | Some Concern | Low | Some Concern | Low | Some Concern | Some Concern |

**Supplementary Table 7. Risk of bias assessment for studies without a comparator group, using the JBI Cohort Study checklist (n = 14)**

| **Author Year** | **Study Design** | **Item 1** | **Item 2** | **Item 3** | **Item 4** | **Item 5** | **Item 6** | **Item 7** | **Item 8** | **Item 9** | **Item 10** | **Item 11** |
| --- | --- | --- | --- | --- | --- | --- | --- | --- | --- | --- | --- | --- |
| **Brown 2020** | Cohort; Retrospective | NA | NA | Yes | Y | NA | Y | Y | Y | N | N | Y |
| **Mahale 2021** | Cohort; Retrospective | NA | NA | U | Y | N | Y | U | Y | N | N | Y |
| **Nath 2003** | Cohort; Retrospective | NA | NA | U | Y | U | Y | Y | Y | N | N | Y |
| **Sarkar 2017** | Cohort; Retrospective | NA | NA | Y | Y | N | Y | Y | Y | Y | Y | Y |
| **Do 2020** | Cohort; Retrospective | NA | NA | U | Y | N | Y | Y | Y | Y | Y | Y |
| **Marois 2017** | Cohort; Retrospective | NA | NA | Y | Y | U | Y | Y | Y | Y | Y | Y |
| **Homma 2020** | Cohort; Retrospective | NA | NA | U | Y | U | Y | Y | Y | Y | Y | Y |
| **Lang 2004** | Cohort; Retrospective | NA | NA | Y | N | N | Y | Y | Y | Y | NA | Y |
| **Malmgren 2011** | Cohort; Retrospective | NA | NA | Y | N | N | Y | Y | Y | Y | NA | NA |
| **Kara 2016** | Cohort; Retrospective | NA | NA | Y | N | N | Y | Y | U | U | U | Y |
| **Wrigley 2025** | Cohort; Retrospective | NA | NA | Y | Y | Y | Y | Y | Y | Y | NA | Y |
| **Tiankinon 2022** | Cohort; Retrospective | NA | NA | Y | Y | Y | Y | Y | U | Y | NA | Y |
| **Goetz 2003** | Cohort; Retrospective | NA | NA | Y | N | NA | Y | Y | Y | N | N | Y |
| **Yamazaki 2011** | Cohort; Retrospective | NA | NA | Y | Y | N | NA | Y | Y | Y | NA | Y |

Y – Yes; N – No; NA – Not Applicable; U - Unsure
